# Giant Cells, Giant Impact: A Case of Aggressive Central Giant Cell Granuloma in the Mandible

**DOI:** 10.7759/cureus.58881

**Published:** 2024-04-24

**Authors:** Akash Doshi, Nitin Bhola, Anchal Agarwal

**Affiliations:** 1 Oral and Maxillofacial Surgery, Sharad Pawar Dental College, Datta Meghe Institute of Higher Education and Research, Wardha, IND

**Keywords:** nerve paresthesia, surgical resection, cortical bone thinning, tooth displacement, root resorption, aggressive central giant cell granuloma

## Abstract

Central giant cell granuloma (CGCG) is a bone lesion characterized by fibrous tissue containing areas of bleeding, giant cells with multiple nuclei, and trabeculae of woven bone. It is considered to be a local bone repair response, possibly triggered by inflammation, bleeding, or local injury. CGCG is more prevalent in females and can occur across a wide age range, typically diagnosed at a young age. Mandibular involvement is more common than maxillary involvement, with most lesions in the posterior region often extending into the ascending ramus. Management of aggressive CGCG can involve non-surgical (medical) and surgical treatment modalities. Surgical approaches vary from simple curettage to en bloc resection depending on various factors discussed in this case report.

## Introduction

Central giant cell granuloma (CGCG) is an infrequent, non-cancerous osseous growth usually located in the mandible and maxilla, accounting for about 7% of all benign tumors in these areas [1]. According to the World Health Organization, CGCG is an osteo-related development inside bone tissue consisting of fibrous cells comprising diverse hemorrhagic areas, clusters of multinucleated giant cells, and infrequent thin plates of new bone formation [2]. Its microscopic appearance is the same as that of brown tumors linked with hyperparathyroidism, emphasizing the need to differentiate between the two conditions through laboratory tests. Despite considerable debate, the exact nature of CGCG remains uncertain [3-6]. CGCG mostly affects the young population, with over 75% of cases seen below the age of 30 years. However, it may appear at any point in time [5]. Females are more frequently afflicted than males, with a proportion of two to one [4,7-11].

According to the research, these types of pathologies in the upper jaw are mostly found in the front, but in the lower jaw, they are more evenly spread between the front and back [11]. In clinical settings, the presentation of CGCG can vary from a slow-developing, painless swelling to a more aggressive form that causes pain. It may lead to local bone damage, root resorption, or teeth displacement [5,12]. Chuong et al. developed a set of standards to distinguish aggressive lesions, encompassing criteria such as discomfort, abnormal sensations, root resorption, swift enlargement, cortical bone penetration, and a high likelihood of recurrence post-surgery [3]. Histopathologically, no precise criteria for distinguishing aggressive and non-aggressive CGCG tumors exist. Nonetheless, the quantity and size of giant cells in comparison to other constituents of the tumor may suggest its clinical behavior [3,13]. Although treatment choices have evolved, there is still no consensus in the literature.

Reports have highlighted medicinal treatments such as alpha interferon, calcitonin, and corticosteroids as potential options, suggesting their possible advantages are worth exploring. Nevertheless, the success rates of medical therapy have not yet matched those of surgery in managing the lesions. When medical therapy fails, additional surgery is often needed [14]. Surgery is regarded as the conventional treatment and continues to be the most widely endorsed approach. Nonetheless, researchers have differing opinions regarding the type of surgery to perform. Curettage excision has been linked with a low recurrence rate for smaller pathologies. In case of recurrence, curettage along with peripheral ostectomy and bone resection is suggested [4]. Unal et al. proposed the use of micro-drilling with a diamond burr in the surgical area to achieve safety margins [15]. En bloc resection, known for its low recurrence rate, is seldom documented in the literature. Only a handful of studies have described this technique, typically followed by reconstruction using an iliac crest graft [16-18].

## Case presentation

**Figure 1:**
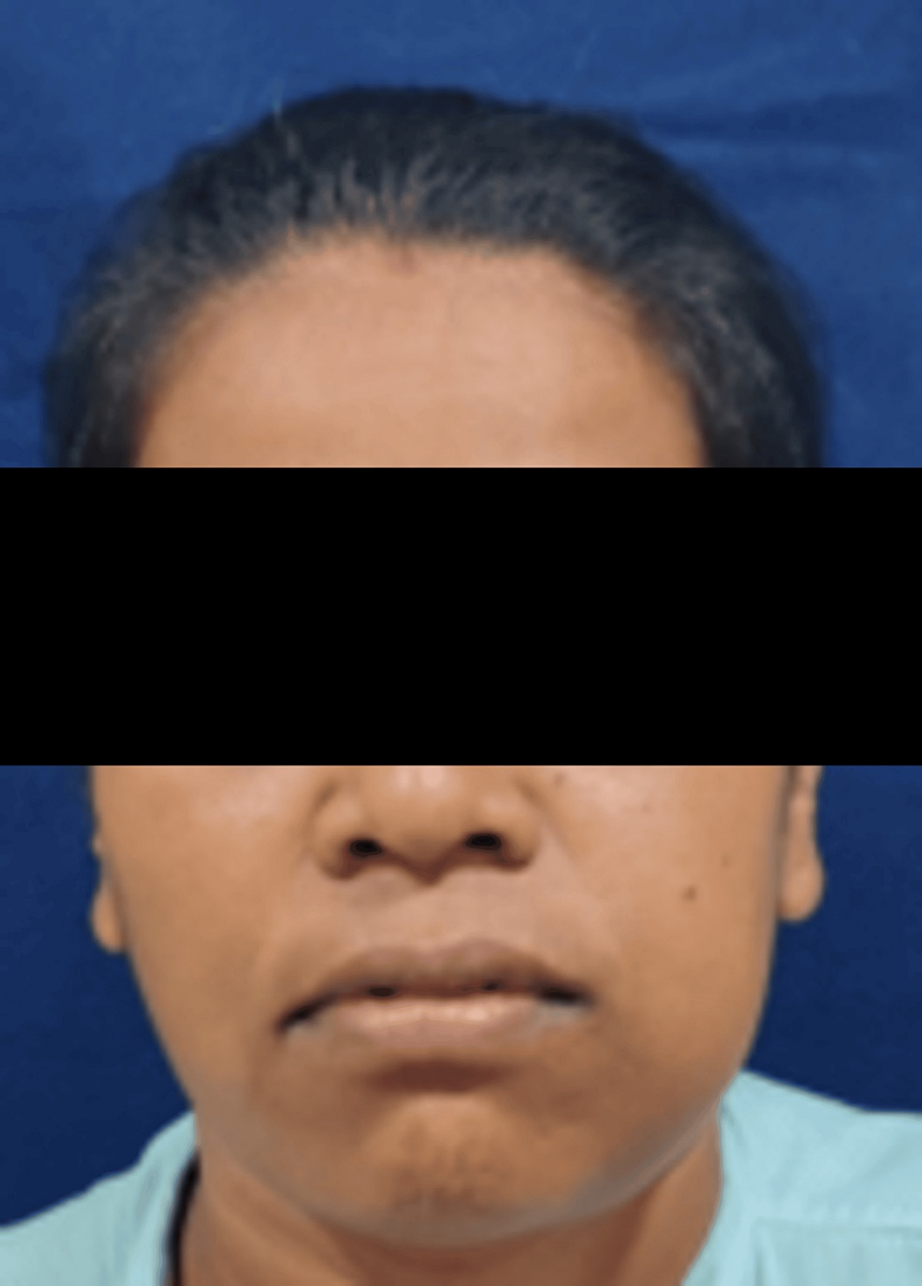
Image of the patient showing swelling over the left side of the face.

**Figure 2:**
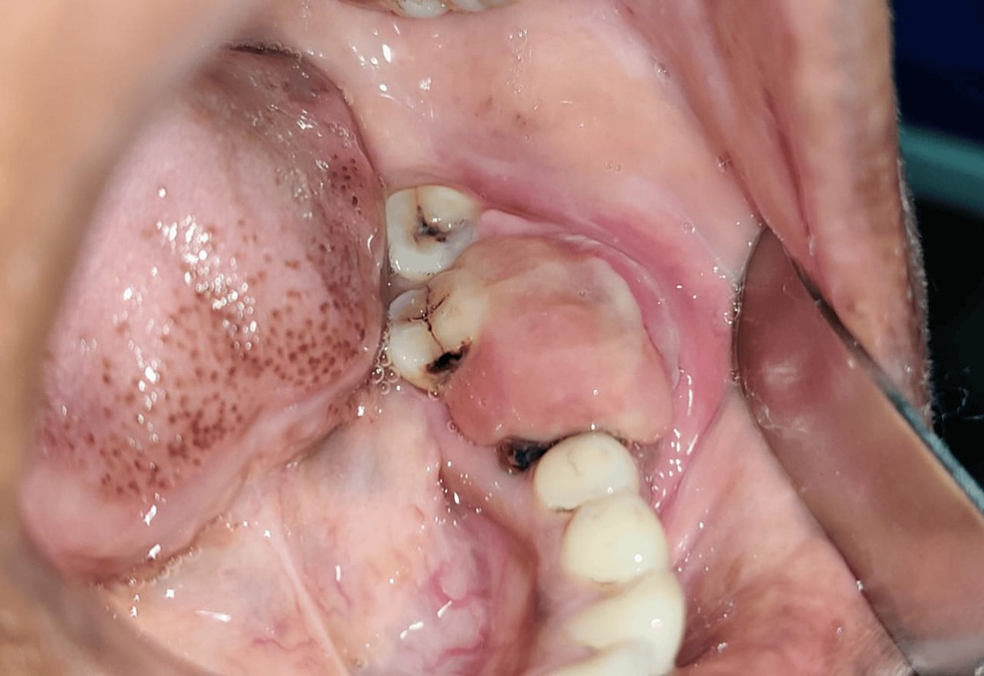
Intraoral image showing the soft tissue lesion over the left posterior region of the jaw.

A 37-year-old woman presented to the Department of Oral and Maxillofacial Surgery, Sharad Pawar Dental College, Wardha, Maharashtra, with a painless swelling on her left cheek, measuring approximately 4 × 3 cm, that had been present for six months (Figure 1). She also reported experiencing nerve paresthesia. Intraorally, she had a 5 × 2 cm swelling involving the left posterior region of the lower jaw, causing difficulty in speech and mastication. During the clinical examination, a significant, diffuse swelling was observed in the left posterior region of the jaw. The swelling had indistinct borders and did not elicit tenderness nor did it exhibit any fluctuation or compressibility (Figure 2).

**Figure 3:**
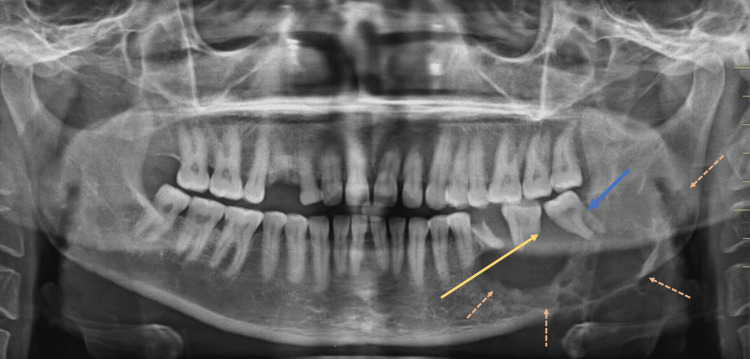
Preoperative orthopantomogram of the patient. Multiple dashed orange arrowheads show the thinning of cortical bone, the yellow arrowhead depicts root resorption, and the blue arrowhead indicates tooth displacement.

**Figure 4:**
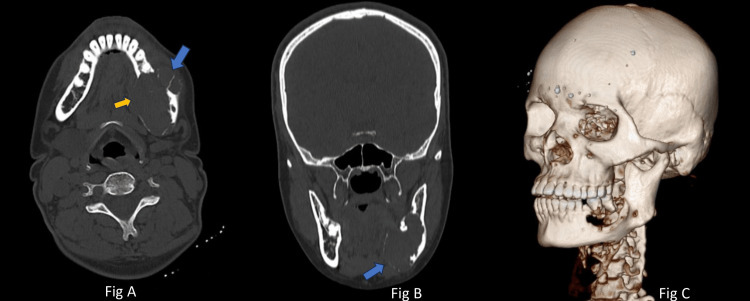
CT scans of the patient. A. Axial section: The blue arrowhead indicates perforation while the yellow arrowhead indicates the thinning of the cortical bone. B. Coronal section: Expansion of both cortical plates. Blue arrowhead: more cortical bone thinning on the lingual side. C. Three-dimensional reconstruction: Root resorption, tooth displacement, and cortical expansion.

An orthopantomogram showed a clearly defined lesion with a mix of radiopaque and radiolucent areas. It extended from the lower left first premolar region, encompassing the angle of the mandible and extending up to the mid-ramus region (Figure 3). On computed tomography (CT), a lesion with soft tissue density was identified in the body of the left hemi-mandible, measuring about 4.5 × 3.8 × 3.8 cm. This caused expansion of the body and ramus of the left hemi-mandible, resulting in thinning of the mandibular cortex. Additionally, mild erosion of the adjacent molar teeth was observed (Figure 4).

The patient’s history, clinical features, and radiological data were used to make a differential diagnosis of ameloblastoma, Pindborg’s tumor, true giant cell lesion, and odontogenic keratocyst. An incisional biopsy was conducted, and histopathology revealed the presence of CGCG.

**Figure 5:**
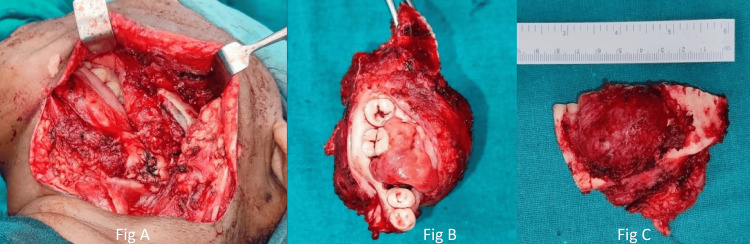
Intraoperative images after tumor resection. A. The image shows the tumor being accessed through a neck (apron) incision, and the tumor removal procedure (segmental mandibulectomy) being performed. B and C: The resected specimen following the surgical removal of the mandible.

Surgical resection of the posterior part of the mandible was planned. Under general anesthesia, the tumor was accessed through an apron incision over the left side of the neck (Figure 5) and segmental mandibulectomy from 34 to the subcondylar region and primary closure was performed.

**Figure 6:**
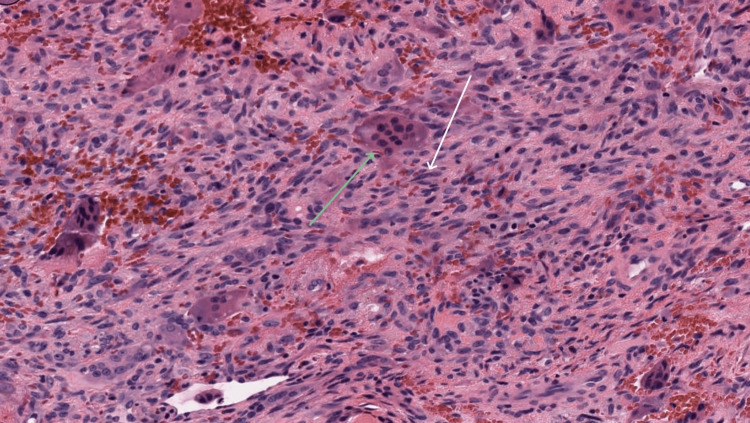
Microscopic picture of the lesion.

Postoperative histopathology findings indicated the presence of mononuclear spindle/polygonal cells (white arrow), along with osteoclast-like giant cells (Figure 6, green arrow) in a vascular background (hematoxylin and eosin, 40×), confirming the diagnosis of CGCG (Figure 6). The patient is currently under observation, and there are no signs of recurrence based on clinical or radiographic assessments.

## Discussion

CGCG displays features of a neoplasm and a reactive proliferative process. Lesions meeting either one major criterion or three minor criteria are categorized as aggressive [3]. Major criteria for this condition include a dimension of 5 cm or more and recurrence following curettage. Minor criteria encompass root resorption, teeth displacement, thinning of cortical bone, perforation, rapid growth, and pain/paresthesia. Generally seen in individuals below the age of 30 years, this condition is more prevalent among women and tends to manifest in the lower jaw rather than the upper jaw. In this case, the patient expressed worries about facial asymmetry caused by a painless, slowly enlarging swelling and numbness on the left side of her face. The lesion was localized to the tooth-bearing area 35-38, involving the left mandibular body and mid-Ramal region, measuring 5 cm × 3 cm.

The orthopantomogram showed evidence of root resorption and tooth displacement, while a CT scan revealed expansion of the mandibular body and ramus, along with thinning of the cortical plates. The radiological characteristics of giant cell granuloma are not distinctly outlined. The lesion can present as a radiolucent area with either a single or multiple compartments, displaying margins that are either well-defined or ill-defined. Additionally, there is often erosion of the cortical plates to varying extents. However, these radiographic appearances are not specific and may be confused with other jaw lesions, such as ameloblastoma or odontogenic keratocyst.

Various treatment approaches have been suggested for managing aggressive CGCG. Curettage alone or combined with resection, with or without loss of continuity, is often the preferred treatment. Some researchers have found favorable results with intralesional injections of corticosteroids. Kaban et al. (2007) [19] recommended a subcutaneous weekly intralesional injection of triamcinolone (10 mg/mL) mixed with a local anesthetic for at least six weeks, along with a daily subcutaneous injection of 1-1.5 million IU of interferon 2a.

Corticosteroids are believed to be effective for CGCG as they inhibit osteoclasts and promote apoptosis, thereby reducing bone absorption. However, their use is associated with disadvantages such as adrenal suppression and should be avoided in patients with diabetes mellitus, peptic ulcer, infection, and immunocompromised states. Schreuder et al. (2017) [20] suggested that treatment with calcitonin injections (100 IU/day subcutaneous or nasal) for 1-1.5 years is an effective alternative to surgery, particularly in cases where vital structures are endangered. Nonetheless, this treatment option is linked to disadvantages such as high cost, requirement for daily administration, and potential side effects such as nausea, vomiting, dizziness, and flushing.

Bredell et al. (2017) [21] proposed using denosumab at a loading dose of 120 mg followed by additional 120 mg doses on days 8 and 15, and then every four weeks subcutaneously. Denosumab can increase mineralization, particularly in small lesions, but it risks medication-related jaw osteonecrosis and impaired healing. Bisphosphonates have been utilized in treating giant cell lesions and fibrous dysplasia in children due to their ability to inhibit osteoclastic bone resorption. One advantage is their supportive action for other forms of treatment. However, they are associated with the risk of osteonecrosis, which depends on factors such as the route of administration, duration of therapy, and additional factors such as past injuries, dental procedures, or inflammation of the teeth and periodontitis.

The management of CGCGs can vary widely, ranging from simple enucleation to radical resection. Surgical approaches are tailored based on factors such as the lesion’s location, size, clinical behavior, and involvement of the periosteum or nerves. As the tumor exhibited aggressive behavior in this case, surgical resection (segmental mandibulectomy) from 34 to the subcondylar region was performed on the left side. Ideal reconstruction options, such as a free fibula graft, were considered for the patient. However, the patient did not opt for free fibula flap reconstruction due to financial constraints. A six-month follow-up revealed no recurrence of the lesion.

## Conclusions

A thorough comprehension of lesions, encompassing their types and their aggressive or non-aggressive nature, is essential for formulating an effective treatment plan for patients. The literature describes various treatment options, encompassing both surgical and medical approaches. The selection of the most suitable surgical method for a highly aggressive tumor, such as CGCG, is crucial. In this instance, the tumor resulted in mandibular thinning and erosion of the outer bone cortex, heightening the risk of a pathological fracture. Consequently, an assertive treatment approach was deemed necessary. Our treatment strategy comprised surgical intervention with segmental resection, followed by appropriate reconstruction.
